# Recent Trends in Cereal- and Legume-Based Protein-Mineral Complexes: Formulation Methods, Toxicity, and Food Applications

**DOI:** 10.3390/foods12213898

**Published:** 2023-10-24

**Authors:** Aprajita Jindal, Nikhil Patil, Aarti Bains, Kandi Sridhar, Baskaran Stephen Inbaraj, Manikant Tripathi, Prince Chawla, Minaxi Sharma

**Affiliations:** 1 Department of Food Technology and Nutrition, Lovely Professional University, Phagwara 144411, India; aprajitajindal0130@gmail.com (A.J.); nikhil2898p@gmail.com (N.P.); 2 Department of Microbiology, Lovely Professional University, Phagwara 144411, India; 3 Department of Food Technology, Karpagam Academy of Higher Education (Deemed to Be University), Coimbatore 641021, India; 4 Department of Food Science, Fu Jen Catholic University, New Taipei City 242062, Taiwan; 5 Biotechnology Program, Dr. Rammanohar Lohia Avadh University, Ayodhya 224001, India; 6 CARAH ASBL, Rue Pal Pastur 11, 7800 Ath, Belgium

**Keywords:** mineral deficiency, inorganic minerals, toxicity, mineral binding, binding efficiency

## Abstract

Minerals play an important role in maintaining human health as the deficiency of these minerals can lead to serious health issues. To address these deficiencies, current research efforts are actively investigating the utilization of protein-mineral complexes as eco-friendly, non-hazardous, suitable mineral fortifiers, characterized by minimal toxicity, for incorporation into food products. Thus, we reviewed the current challenges in incorporating the cereal-legume protein-inorganic minerals complexes’ structure, binding properties, and toxicity during fortification on human health. Moreover, we further reviewed the development of protein-mineral complexes, characterization, and their food applications. The use of inorganic minerals has been associated with several toxic effects, leading to tissue-level toxicity. Cereal- and legume-based protein-mineral complexes effectively reduced the toxicity, improved bone mineral density, and has antioxidant properties. The characterization techniques provided a better understanding of the binding efficiency of cereal- and legume-based protein-mineral complexes. Overall, understanding the mechanism and binding efficiency underlying protein-mineral complex formation provided a novel insight into the design of therapeutic strategies for mineral-related diseases with minimal toxicity.

## 1. Introduction

Micronutrient deficiencies are prevalent globally, affecting almost every age group and causing severe negative health effects [1]. Iron, zinc, calcium, magnesium, and copper are particularly deficient in pregnant women and children under 5 years of age [2]. These essential nutrients are regulated by dietary habits, food intake, mineral absorption, and recycling in the human body [3]. Mineral deficiency can cause severe diseases and disorders, being directly or indirectly associated with physiological challenges such as inadequate tissue oxygen delivery, weakness, cognitive impairment, decreased productivity, and an increased susceptibility to infections [4].

Likewise, iron deficiency can lead to improper cell signaling, mitochondrial respiration, and intermediary metabolism, while zinc deficiency can cause congenital anomalies, prenatal growth retardation, an impaired immune system, and cognitive impairment [5]. Calcium deficiency is a major problem in children and old age populations and it can affect nerve transmission, vascular contraction, intracellular signaling, and hormonal secretion in the human body [6]. Magnesium deficiency has been linked to various health issues such as cardiovascular, respiratory, and neurological problems. The insufficient intake of this mineral can disrupt glucose and insulin metabolism, influence lipid profiles, and reduce inflammatory processes [7]. Copper deficiency can affect other vital processes in the human body, including energy metabolism, reactive oxygen species detoxification, and iron uptake [8].

In recent years, the significance of micronutrient deficiency has been increasingly recognized by government, non-government, and industrial officials. However, diversifying diets to tackle mineral deficiencies is not always feasible due to factors such as affordability, availability, and religious constraints [9]. Additionally, certain foods contain anti-nutritional properties that can inhibit the absorption of minerals, leading to deficiencies in the human body [10]. Therefore, food fortification via external mineral sources has been identified as an effective approach by the World Health Organization (WHO) and the Food and Agricultural Organization (FAO) to utilize the existing food industry distribution system [11].

Several inorganic salts have been added to the food as a source of minerals, which are responsible for various factors such as bioavailability, chemical stability, appearance, and uniformity of the fortified food product [12]. For many years, these inorganic salts have served as an alternative in combating mineral deficiencies by offering a sustainable and economical approach to the nutritional security of a large population [13]. However, in recent studies, inorganic minerals have also shown several disadvantages due to their toxic effects, which raised major concerns about severe impacts on human health [14]. The studies revealed that the long-term use of inorganic minerals leads to toxicity due to their accumulation in the human body. The effect of toxicity is characterized by the accumulation at the tissue level ranges from mild (nausea and abdominal pain) to severe (liver abnormalities and increased intracranial pressure) in the human body [15]. Additionally, the presence of inorganic minerals in free form causes extreme toxicity due to their ability to generate free reactive oxygen species, whereas their long-term use in excessive amounts also causes metabolic acidosis [16]. The prolonged use of calcium carbonate leads to nephrotoxicity and hypercalcemia, and in pregnant women, fetotoxicity has been observed [17]. The deposition of free zinc ions in humans leads to neurotoxicity due to their accumulation in the brain [18]. Also, the consumption of inorganic minerals of calcium has shown toxic effects leading to gastric necrosis along with milk-alkali syndrome and alkalosis affecting human health [19]. Therefore, recent studies have addressed the effective use of different interactions to bind the mineral-protein and thereby form a complex with these inorganic minerals [20].

This review focuses on the efficiency of cereal- and legume-derived protein-mineral complexes with a particular focus on the importance of conducting in vitro, in vivo, and potential toxicity studies. Compared to the published works, this review highlights the development of protein-mineral complexes and various characterization techniques used to study the binding ability, including chemical interactions that have been involved. Furthermore, the review highlights the potential functional benefits of using cereal- and legume-based protein-mineral complexes in improving mineral bone density and antioxidant activity. Overall, this review provides valuable insights into the interactions between minerals and cereal-legume-derived proteins, offering a potential pathway toward developing sustainable and safe mineral fortificants for a diverse range of food products.

## 2. Cereal and Legume Proteins

Cereals are the edible grains or seeds that belong to the Gramineae genus of grass. Wheat and rice are of the most significance on a global scale as it accounts for more than 50% of the world’s cereal production [20,21]. In both developed and developing nations, cereals serve as a staple food regardless of their cultures and beliefs. Worldwide, legumes belonging to the family Fabaceae are the second most popular food crop after cereals, thereby serving as a vital source of food for the poor living in developing and under-developing countries. Furthermore, this paper discusses two key sources of protein, specifically “cereals” and “legumes”.

### 2.1. Cereal Proteins

The edible seed of several cereal grains is distinguished from one another based on their shape, size, mass, and other characteristics. There are different components of a cereal grain, namely endosperm, bran, aleurone layer, and germ [21]. The endosperm is the largest morphological component and stores nutritive compounds such as starch and proteins [22]. In cereal and pseudo-cereal grains, bran is the hard outer layer consisting of pericarp and inner seed coats rich in dietary fiber, protein, and fat. The third component is the aleurone layer which is the outermost layer of the endosperm and varies among different grains in terms of thickness. Studies have reported two types of aleurone cells in the layer: the first one is cuboidal-shaped, which covers the outer part of the endosperm, whereas the second type, also called the modified aleurone layer, functions in surrounding the embryo [23]. Finally, the germ is a by-product of cereals consisting of a majority of nutrients such as high-quality essential amino acids, lipid content, and other nutrients [24].

In general, the chemical composition of cereals includes carbohydrates (65–75%), proteins (7–12%), lipids (2–6%), and certain minerals (1.5–2.5%). Protein is the second-largest component of cereal grains, which is of great importance due to its functionality and the availability of different amino acids with varying molecular weights [25]. A systemic study on the plant protein was carried out by “Thomas Osborne”, known for his work towards the classification of plant proteins based on their solubility in different solvents, and ‘cereal grain’ was the first to be studied for their protein classification [26]. Moreover, they are categorized into four types depending on their solubility, namely albumins (soluble in water), globulins (soluble in a salt solution), prolamins (soluble in ethanol aqueous solution), and glutenins (dilute acid or alkaline solution). Different types of cereal grains such as wheat, rice, maize, barley, and oats consist of a variety of proteins that are discussed in more detail [27]. For instance—wheat consists of a storage protein called gluten (80–85%) which divides into two subgroups, gliadins (alcohol soluble) and glutenins (alcohol insoluble). Gliadins and glutenins are composed of distinct structures, each with different molecular weights and amino acid contents [28]. In gliadins, α, β, and γ categories of monomeric proteins are present with a molecular weight of 30–45,000 Da and contain the amino acids glutamine (30–40%), proline (15–20%), cysteine (2–3%), and lysine (<1.0%), whereas ώ gliadins are also the monomeric type of proteins but have a different molecular weight, i.e., 40–75,000 Da containing the amino acids glutamine (40–50%), proline (20–30%), phenylalanine (8–9%), and lysine (0–0.5%). On the other hand, glutenins are polymeric proteins with high molecular weight (65–90,000%) subunits, low molecular weight (30–45,000%) subunits, and exceptionally high levels of glutamine and proline [29]. Along with this, it also contains metabolic proteins such as albumin, globulin, and amphiphilic proteins. Albumins and globulin are suggested to have better amino acid content due to high levels of methionine and lysine. The rice protein, which is hypoallergenic and highly acceptable for consumption, consists of protein content that varies with its types of milling fractions such as paddy (5.6–7.7%), hulled rice (2.0–2.8%), brown rice (7.1–8.3%), rice bran (11.3–14.9%), and milled rice (6.3–7.1%). Solubility and molecular weight are the two criteria by which rice proteins are divided into four groups. The albumins protein is water soluble and is present in the endosperm (3.8–8.8%) and the bran layer (35%) of rice with its molecular weight in a range of 14–16 kDa. Globulin is a salt-soluble protein that is present in endosperm up to 9.6–10.8% and about 15–26% in the bran layer of rice. These are further classified into four types α, β, γ, and ώ having a different range of molecular weight. The amino acid content for globulin is higher in cysteine and methionine but lower in lysine content. Prolamins are soluble in alcohol and comprise protein up to 2.6–3.3% in the endosperm and 4% in the bran layer. Additionally, studies showed three subunits of the polypeptide with varying molecular weights, i.e., 10, 13, and 16 kDa were also identified. In terms of amino acids, it contains a high level of glutamine, glycine, alanine, and arginine (Eakkanaluksamee and Anuntagool, 2020). Lastly, glutelins which are readily soluble in acidic (pH < 3.0) and alkaline conditions (pH > 10.0) and function as major storage proteins in rice are present in the endosperm up to 66–78% and up to 11–27% in the bran layer. They are composed of two polypeptide subunits, namely acidic and basic, with molecular weights of 30–39 and 19–25 kDa [30]. Maize, which is known as the queen of cereals, generally consists of poor-quality protein. The protein content of maize is up to 10% and they are classified into four categories based on their solubility: albumins (3%), globulins (3%), glutelins (34%), and prolamins (>60%) [31]. Prolamins are called zeins and are further classified into four types, having a wide range of molecular weights, α (19 & 22 kDa), β (15 kDa), γ (50, 27 & 16 kDa), and δ (18 & 10 kDa). These proteins contain different types of amino acids. For example, β-zein is characterized by high levels of cysteine and methionine, δ-zein is rich in methionine, γ-zein contains cysteine, while α-zein lacks all essential amino acids. Barley (*Hordeum vulgare*), a cereal grain, consists of four different proteins: albumins, globulins, glutelins, and prolamin. Prolamin is divided into another class of protein called hordeins containing 35–45% protein and is further classified into different fractions with each having a specialty. B-hordeins, serving as the major storage protein, coexist with C-hordeins, comprising approximately 10–20% protein content. D-hordeins exhibit a substantial molecular weight exceeding 100 kDa, while γ-hordeins are characterized by their abundance in sulfur-rich amino acids [32]. Oat is a highly protein-rich cereal and its content is distributed throughout its structure: endosperm (12%), bran (18–26%), and germ (29–38%). They are further classified into four categories: globulins, prolamins, albumins, and glutelins. Globulins, consisting of 70–80% of protein, are major storage proteins and are soluble in the salt solution. Additionally, it consists of a 12S subtype which has been reported to consist of 74% of β-sheets and its molecular weight ranges up to 53–58 kDa. The other two subtypes are 7S and 3S, which are also storage proteins with different molecular weights ranging from 50–70 kDa and 48–52 kDa. Oat prolamins, called avenins, consist of high levels of glutamic and glutamine amino acids. Avenins have a molecular weight of 17–34 kDa and a protein content of up to 4–14%. Albumins, a metabolic protein, contains a rich source of lysine amino acid. It has a molecular weight in the range of 14–17, 20–27, and 36–47 kDa with 1–12% of protein content. Glutelins, which are soluble in dilute acidic and alkaline conditions, contain a protein content of less than 10%.

### 2.2. Legume Proteins

In legume seeds, the structures of the protein are reported to have a high content of β-sheets and a comparatively low content of α-helix. Legumes have two major components, namely the seedcoat and the embryo. The structure and composition of seed coats vary among different species of legumes [33]. It functions in transferring information related to the external environment while also protecting the embryo. Moreover, the embryo contains two cotyledons, namely radicle and plumule, and it functions in enclosing the embryo axis while also providing a source of food storage for its development. The chemical composition of legumes includes proteins (20–45%), along with essential amino acids, dietary fiber (5–37%), and complex carbohydrates (up to 60%). In addition to this, it also contains certain minerals and vitamins and is generally low in fat content with no cholesterol levels, making it a nutritious rich crop [34]. According to the new classification system, legumes are classified into three sub-families which include Papilionoideae, Caesalpinioideae, and Mimosoideae. The major variety of edible legumes is covered under Papilionoideae such as peanut, chickpea, soybean, common bean, clover, mung bean, lentils, lupins, faba beans, vetches, and peas [35]. According to the studies, different species of legumes have a wide range of proteins based on their amino acid composition and molecular weight. Chickpeas consist of 17–22% protein which is divided further into globulins, albumins, glutelins, prolamins, and some residual proteins [35,36]. Globulins are the main storage proteins consisting of 56 g per 100 g of protein. They comprise two types of protein, which are 11S legumin and another 7S vicilin, having a molecular weight range of 320–400 kDa and 145–190 kDa, respectively [36]. Albumins are richer than globulins as they contain more essential amino acids such as tryptophan, lysine, and threonine, and also these fractions include more enzymatic and metabolic proteins. Glutelins are structurally similar to globulins as they belong to the 11–12S family of globulins, whereas prolamins are reported to be found in trace amounts irrespective of the chickpea variety. The soybean protein is utilized by consumers in various forms and it generally contains about 70% of total protein [37]. Studies have revealed that the soybean protein is called a complete food as it consists of all nine essential amino acids and is highly nutritious [38]. They are further divided into two types of storage proteins which are 11S (globulins, glycinin) and 7S (β-conglycinins). The 11S (globulins, glycinin) protein is a hexameric complex having a molecular weight in the range of 320–275 kDa, whereas 7S (β-conglycinins) further divides into three sub-units (β, α, and α’) having 50, 60, and 71 kDa size of molecular weights [33]. The amino acid composition of soybean contains histidine, isoleucine, leucine, lysine, methionine, phenylalanine, threonine, tryptophan, and valine [39]. Mung bean contains approximately 85% of total proteins which contribute majorly to the storage of proteins in the mung bean [40]. It includes three types of proteins based on their solubility, albumins (soluble in water), globulins (soluble in dilute saline), and prolamins (soluble in alcohol-water mixtures). Different sub-types, namely 7S (basic type), 8S (vicilin type), and 11S (legumin type) of globulin, are also present [40,41]. Molecular weight also varies among sub-types, such as 8S having a range of 26–60 kDa, 7S having 28 and 16 kDa sizes, and 11S having 40 and 24 kDa sizes. Globulins and albumins consist of 60% and 25% of protein from the total content of mung bean protein. Moreover, mung bean is rich in certain essential and aromatic amino acids such as leucine, isoleucine, valine, and glutamic acid although they are deficient in sulfur-containing amino acids such as methionine and cysteine along with tryptophan [41]. Peas contain 20–25% protein and pea germplasm, which is a part of pea seed, has also been reported to have a protein content of approximately 30%, although it differs based on genotypes and environmental factors [42]. Moreover, it can be classified into four types: globulin, albumin, prolamin, and glutelin. Globulins are soluble in the salt solution and contain 55–65% of total protein and are further divided into sub-types, namely 11S legumin and 7S vicilin. Legumin (11S) is a hexameric protein whereas vicilin (7S) is a trimeric protein with both having a molecular weight in the range of 320–400 kDa and 150–180 kDa, respectively [36]. The amino acid varies in both the sub-types as it has more sulfur-containing amino acids in the legumin type per unit of protein. Albumins have 18–25% of total protein and are classified into two small molecular weight albumins, namely PA1a and PA1b, with a range of 6 kDa and 4 kDa in size, respectively, and they have also shown a high content of cysteine amino acid. Further, prolamin and glutelin are also a group of storage proteins, but they present only small amounts of protein in pea seeds. Also, the pea protein is identified as a protein having a well-balanced amino-acid profile since it contains high levels of lysine, leucine, and phenylalanine [43].

Therefore, plant-based proteins have a greater scope in replacing animal-based proteins since they have a huge potential in providing a sustainable approach. Moreover, the choice of a protein source is equally essential since its structure and side chains decide the efficient binding of minerals, thereby leading to the preparation of protein-mineral complexes. Also, new applications can be focused on by using these high-potential legume- or cereal-based proteins to overcome the problem of toxicity by binding them with inorganic minerals.

## 3. Development of Protein-Mineral Complexes

**Figure 1:**
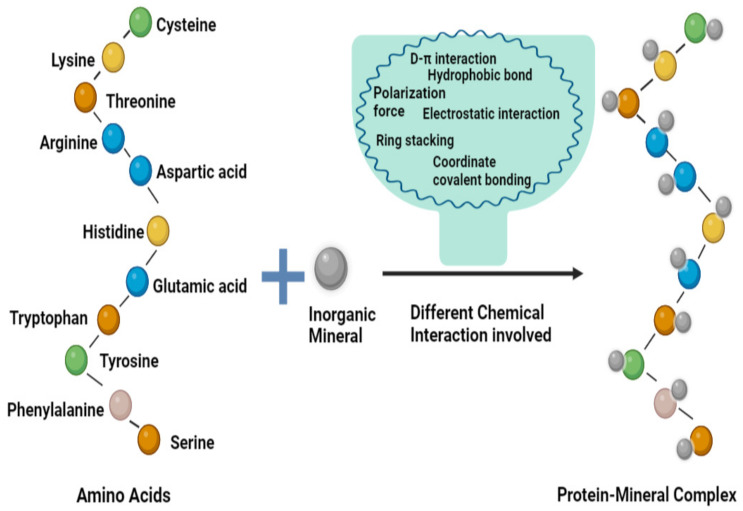
Chemical interactions are involved in the preparation of cereal and legume protein-mineral complexes. Figure is adapted from Sun et al. [51] and is an open-access article (copyright ©2020 by authors) distributed under the terms and conditions of the Creative Commons Attribution (CC BY) license.

In recent years, there has been significant research focused on the nutritional function and bioactivity of proteins and peptides. One approach that has gained considerable attention is the study of proteins with the capacity to bind minerals, as they have been found to enhance the bioavailability of minerals upon consumption [44]. Moreover, various types of chemical interaction are involved during the formulation and stabilization of protein-mineral complexes [45]. The amino acids present in proteins have side chains that consist of binding sites and they interact with inorganic minerals to form a complex. Some major groups that have been shown to bind to inorganic minerals are the imidazole functional group of histidine, the aromatic benzene ring of tyrosine, the sulfhydryl functional group of cysteine, the β carboxyl group of aspartate, and the γ carboxyl group of glutamic acid [46]. These side chains are highly exposed and provide functional binding sites, thus helping with the interaction between the metal ion (electron receptor) and ligand (electron donor), and they also function in stabilizing the protein-mineral complexes (Figure 1) [47]. Moreover, these functional groups are classified into three categories, namely non-coordinating (alanine, valine, leucine, isoleucine, phenylalanine, and tryptophan), weakly coordinating (serine, threonine, tyrosine, lysine, arginine, aspartic acid, glutamic acid, asparagine, glutamine, and methionine), and strongly coordinating (histidine and cysteine) side chains [48]. Inorganic minerals also require several specific properties to interact with proteins, such as their size and specific electronic configuration. The different types of chemical interactions involved in the binding and chelation mechanism are electrostatic, hydrophobic, hydrophilic, ionic, n-π*, hydrogen bonding, coordinate covalent bonding, coordination, and weaker interactions [49,50].

In this context, Table 1 reveals different interactions among cereal/legume-based protein-mineral complexes. For instance, in a study of a peanut protein interaction with the zinc mineral, the binding efficiency increased due to the n-π* interaction between the protein and mineral ion due to the formation of the coordinate covalent bond with hydroxyl oxygen, carboxyl oxygen, and amino nitrogen atoms present in the peptides with the zinc minerals [52]. Another study was based on the *Phaseolus vulgaris* L. protein with the copper mineral where their interaction was found to be influenced by both electrostatic and ionic forces. During the interaction, copper showed binding to the thiol group of amino acids, and the presence of the carboxylic group also provided binding sites to the copper minerals, leading to high affinity [53]. The interaction of the barley protein with different inorganic minerals (iron, calcium, copper, and zinc) was also studied. It was shown that the presence of weakly and strongly coordinated side chains in a higher content such as Asx (asparagine + aspartic acid), Glx (glutamine + glutamic acid), cysteine, and lysine lead to effective binding to these inorganic minerals with the help of electrostatic or coordination interaction [54]. Several studies on chelation were also conducted—for instance, the oat bran protein with iron and calcium minerals was chelated via electrostatic and ionic interaction between them due to the negatively charged carboxylic moieties having an excess of electrons, which enhanced the protein chelation with minerals [55,56].

Another study showed the chelation ability of iron minerals to the soy protein via the presence of oxygen-rich groups of carboxyl and phosphate in two amino acids (aspartic and glutamic) which chelated iron minerals via electrostatic interaction [57]. Rice bran protein chelation was also studied with copper minerals, which showed due to the presence of several amino acid groups such as polar-uncharged amino acids (serine and cysteine), sulfur-containing amino acids (cysteine and methionine), the guanidine group of arginine participate in hydrogen bond formation, and the interactions that occurred via electrostatic force [58]. The interaction capacity of the wheat germ protein with the zinc mineral was observed due to the electrostatic interaction between them with the presence of an imidazole ring of Histidine at the N terminal being majorly responsible for binding the zinc mineral [59]. In a study, both the carboxyl group and imidazole group via electrostatic force showed the interaction between the soy protein with calcium minerals [60]. The binding of the mung bean protein to iron minerals was due to the presence of both hydrophobic (leucine, isoleucine, valine, and proline) and hydrophilic (aspartic acid, glutamine, arginine, and lysine) amino acids in the peptide, leading to a higher binding of inorganic minerals due to the hydrophobic and hydrophilic interaction [51]. In a study on the red lentils and iron interaction, the electrostatic force between the amino acids of the protein and mineral has shown an increase in the binding via the tendency of the carboxylic or amine group to deprotonate the electron-donating groups at pH 7 [61]. The sesame protein interaction with the zinc mineral showed binding due to several functional groups present in amino acids such as the carboxyl group, the amino group of peptides, the sulfhydryl group of cysteine, the hydroxyl group of serine via coordination interaction, along with a weaker interaction in which the carbonyl or amino group with water molecules have shown to interact with the zinc minerals [62]. The black bean protein was also studied for its interaction with calcium minerals and it showed the presence of two acidic groups (aspartic and glutamic) of amino acids which deprotonated and acquired a negative charge, thereby enhancing their interacting capacity [63]. Also, histidine with the imidazole group was present in high amounts in the black bean protein, which showed the interaction with minerals by providing binding sites [64]. In these complexes, the presence of hydrophilic amino acids such as lysine, arginine, serine, and threonine was also observed to have shown interaction with mineral ions, thereby increasing the binding ability.

Overall, the study of protein-mineral complexes has gained significant attention in recent years due to their ability to enhance the bioavailability of minerals. The amino acid side chains present in proteins interact with inorganic minerals via several types of chemical interactions, such as electrostatic, hydrophobic, hydrophilic, ionic, n-π*, hydrogen bonding, coordinate covalent bonding, and weaker interactions. Different types of cereal/legume-based protein-mineral complexes have been studied, and the interactions that take place among them have been reviewed. These studies have shown that the binding efficiency between proteins and inorganic minerals is influenced by the properties of the minerals and amino acids in the proteins. Therefore, these studies have important implications for improving the nutritional value of foods by enhancing the mineral bioavailability via the development of protein-mineral complexes.

**Table 1:** Interaction between various cereal/legume proteins and minerals.

Mineral	Source of Protein	Type of Interaction	References
Iron	Red Lentil Protein Hydrolysate	Electrostatic interaction	[48]
Iron	Mung-Bean Protein Hydrolysate	Hydrophobic interaction	[37]
Calcium	Soy Bean Protein Hydrolysate	Electrostatic interaction	[65]
Iron	Mung Bean Protein Hydrolysate	Hydrophobic and hydrophilic interaction	[52]
Iron	Mung Bean Protein Hydrolysate	Covalent interaction	[32]
Iron	Soybean Protein Hydrolysate	Electrostatic interactions	[36]
Iron	Soy Protein hydrolysate	Electrostatic interaction	[34]
Copper and Iron	Jamapa Protein Hydrolysate	Electrostatic and ionic interaction	[55]
Zinc	Peanut Protein Isolate	n-π* interaction	[39]
Zinc	Sesame Protein Hydrolysate	Coordination and weaker interactions	[41]
Cadmium	Rice Protein	Coordination interaction	[55]
Iron, Calcium, Copper, and Zinc	Barley Protein Hydrolysate	Electrostatic interaction and hydrophobic interaction	[43]
Calcium	Black Bean Hydrolysate	Electrostatic and hydrophilic interaction	[43]
Iron	Oats Protein Hydrolysate	Electrostatic interaction	[34]
Calcium	Wheat Germ Protein Hydrolysate	Electrostatic interaction	[48]
Copper	Rice Bran Protein	Electrostatic interaction	
Calcium	Sunflower seed protein	Ionic interaction	[55]
Calcium	Peanut seed protein	Ionic interaction	[55]

### 3.1. Binding Efficiency and Characterization of Cereal- and Legume-Based Protein-Mineral Complexes

#### 3.1.1. Binding Efficiency of Complexes

The binding efficiency of protein-mineral complexes refer to the strength and extent of binding between a peptide and a mineral. The binding efficiency can be quantitatively measured by determining the binding affinity, stoichiometry, and kinetics of the peptide-mineral complexes [65]. Peptide-mineral binding interactions are studied using a variety of techniques, each with its advantages and limitations, and the choice of technique depends on the specific properties of the peptide and mineral being studied. The techniques involved in studying protein-mineral binding is diverse and encompass both experimental and computational methods. Moreover, these techniques were used to study the binding efficiency of various cereal- and legume-based protein-mineral complexes (Table 2). An example of a study on the peanut protein demonstrated that the peptide’s spatial structure of the peanut protein played a significant role in its binding capacity to calcium. Each fraction of the peanut protein showed different calcium-binding capacities, with the PP1 fraction (≥10 kDa) exhibiting the highest binding activity of 124.7 mg/g. The study also revealed that calcium is bound to the peptide of the peanut protein via various amino groups, including amide and carboxyl groups on the side chain of the peptide [66]. After the purification of the Mung bean protein hydrolysate using anion exchange chromatography, the binding capacity of the protein with iron increased significantly. The highest binding efficiency was observed in the AF2 fraction, which was found to be rich in Glx (Glu + Gln), Asx (Asp + Asn), Leu, Lys, Arg, Phe, and Ser. The AF2 fraction had a higher content of negatively charged amino acids, which increased with a longer retention time on the column (AF1–AF4) and corresponded to an increase in acidic amino acids (Glx and Asx). Overall, these findings suggest that the AF2 fraction has a superior iron-binding capacity compared to other fractions [67]. The medium-sized peptide fraction (1–5 kDa) of the barley protein hydrolysate showed the highest binding capacity for calcium, iron, copper, and zinc minerals. The binding was attributed to the presence of amino acids such as Asx (Asp + Asn), Glx (Glu + Gln), Cys, and Lys, which have reactive side chains capable of forming electrostatic or coordination interactions with the minerals [68]. The highest binding capacity was observed for copper (133.53 µg/mL), followed by zinc (125.2 µg/mL), iron (87.16 µg/mL), and calcium (14.3 µg/mL) [54]. The wheat germ protein and zinc mineral complexes exhibited a notable binding efficiency of 91.67%. This strong binding was attributed to the electrostatic interaction between the protein peptides and the mineral, as well as some degree of coordination interaction. Similarly, another study on the wheat germ protein hydrolysate and its binding with calcium minerals demonstrated that the maximum calcium bound to the protein was 67.5 mg/g. It was found that the binding capacity was influenced by various factors, including the type of enzyme used, degree of hydrolysis, amino acid composition, and molecular mass distribution. The wheat germ protein peptide fragment that exhibited the highest binding capacity was attributed to the presence of key amino acids (such as Glu, Asp, Arg, and Gly), had a molecular mass of less than 2000 Da, was hydrolyzed via the Alcalase enzyme, and had a degree of hydrolysis of 21.5% [69]. The study of cadmium binding to rice proteins showed that the maximum binding capacity was 15.26 mg/g, with a strong affinity between the two due to the morphological characteristics of the rice protein [70]. Specifically, the protein was found to have a significant number of coordination sites on its surface, leading to a multidentate coordination pattern. The binding process was observed to be rapid and endothermic [71].

Overall, the binding efficiency of protein-mineral complexes is determined via various factors, including the spatial structure of the protein, amino acid composition, molecular mass distribution, and the morphological characteristics of the protein-mineral complexes [72]. Various techniques are available for studying these interactions, ranging from experimental to computational methods. Studies on cereal- and legume-based protein-mineral complexes have shown that certain protein fractions exhibit higher binding capacities for specific minerals, with the binding attributed to the presence of amino acids capable of forming electrostatic or coordination interactions with the mineral. Understanding the binding efficiency of protein-mineral complexes can have implications in developing functional foods and supplements with enhanced nutritional value.

#### 3.1.2. Characterization Using Various Analytical Techniques

Experimental techniques include IMAC (ImmoTbilized Metal Ion Affinity Chromatography), RP-HPLC (Reverse Phase High-Performance Liquid Chromatography), isothermal titration calorimetry (ITC), SD-PAGE (Sodium Dodecyl Sulfate Polyacrylamide Gel Electrophoresis), Fourier Transform Infrared Spectroscopy (FTIR), and mass spectrometry. These methods allow us to visualize the three-dimensional structure of the peptide-mineral complexes, measure the binding affinity and kinetics, and identify specific amino acid residues involved in the binding process.

**Figure 2:**
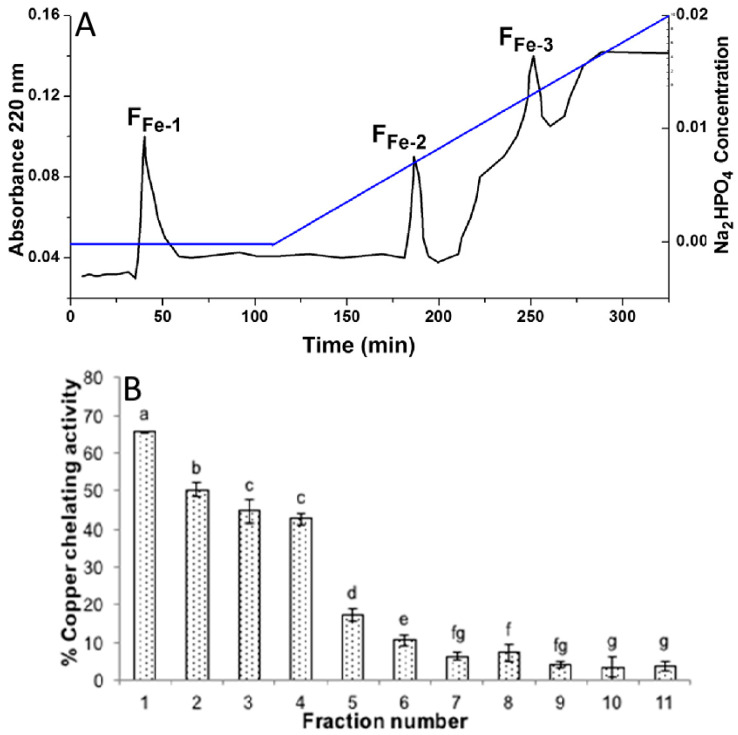

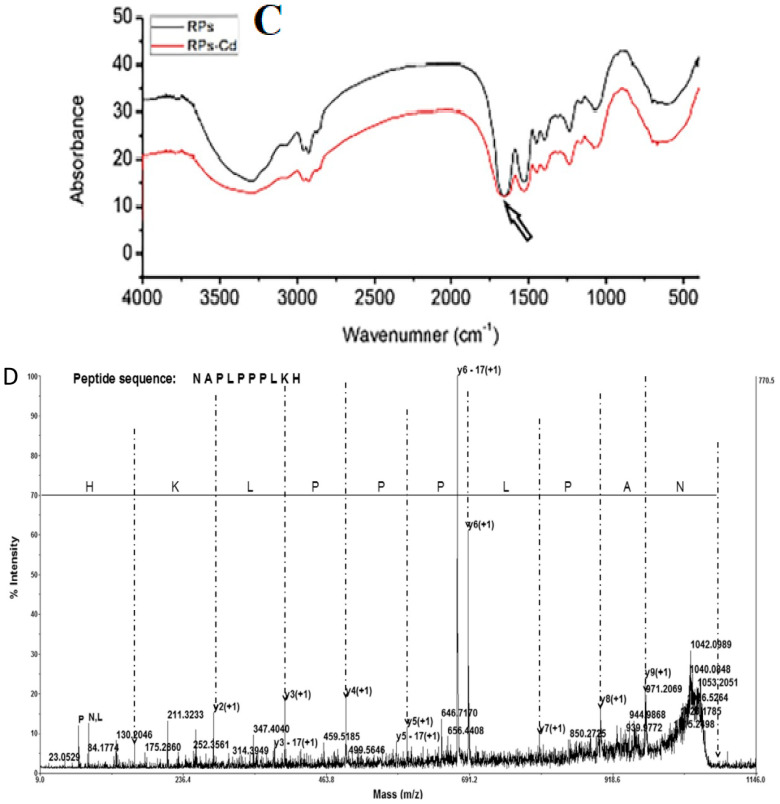
(**A**) Immobilized metal affinity chromatography (IMAC-Fe^3+^) graph; subfigure is adapted with permission (copyright ©2013 Elsevier B.V., Amsterdam, The Netherlands) and from Lv et al. [75]. (**B**) Copper-chelating activity of rice bran albumin hydrolysate (RBAIbH) fractions at 1 mg/mL protein concentration; subfigure is adapted with permission (copyright © 2018 American Chemical Society, Washington, DC, USA) from Kubglomsong et al. [76]. (**C**) FTIR of rice protein and their protein-cadmium complexes; subfigure is adapted with permission (copyright ©2018 Elsevier B.V., Amsterdam, The Netherlands) from Feng et al. [77]. (**D**) Identification of the zinc-chelating peptides via MALDI-TOF/TOF; subfigure is an open access article (copyright ©2014 Elsevier B.V., Amsterdam, The Netherlands) from Ke-Xue et al. [78] and is distributed under the terms and conditions of the Creative Commons Attribution (CC BY) license.

IMAC is a technique used to analyze the binding of protein to minerals. This technique relies on the specific interaction between inorganic mineral ions immobilized on a solid support and protein residues, such as histidine, that have an affinity for inorganic mineral ions. Further, the protein-mineral complexes are loaded onto the column and allowed to pass through. Protein with a high affinity for the immobilized inorganic mineral ion binds to the column, while other components pass through. The column is then washed to remove any unbound proteins, and the bound proteins are eluted using a buffer solution that disrupts the protein-mineral interaction. Moreover, IMAC analysis provides a highly selective and efficient way to isolate any analyzed protein-mineral complexes, which provides valuable information about the binding affinity and mechanism of these complexes. For instance, a study conducted to obtain the soybean protein hydrolysate (3–10 kDa) with calcium-binding capacity fractionation was performed to assess the properties of the protein on IMAC-Ca^2+^ and IMAC-Fe^3+^ columns, respectively. Furthermore, it demonstrated that the fractions from IMAC-Fe^3+^ showed higher calcium-binding abilities and it also showed that the calcium-binding capacity of the protein was correlated with their adsorption ability on the affinity column (Figure 2A) [73]. Thus, the F_Fe-1_, F_Fe2_, and F_Fe3_ fractions obtained from the IMAC-Fe^3+^ column were further evaluated for their other different properties [74].

RP-HPLC is a powerful technique for analyzing protein-mineral binding interactions. RP-HPLC separates molecules based on their hydrophobicity, which is influenced by their interaction with a mineral surface. In RP-HPLC analysis, a sample containing the protein-mineral complexes is loaded onto a column packed with a stationary phase that has a hydrophobic surface. For instance, the copper-chelating activity from the hydrolyzed rice bran-derived protein showed during RP-HPLC that the fractions that were eluted earlier showed the greatest chelating activity. In addition, the result suggested that the RBAIbH fraction showed a stronger copper-chelating activity (Figure 2B) [79].

Isothermal titration calorimetry (ITC) is another useful technique to study the thermodynamics of peptide-mineral binding interactions. ITC measures the heat changes associated with the binding process between a peptide and a mineral. The amount of heat released or absorbed is directly proportional to the amount of peptide that binds to the mineral surface. ITC allows the determination of the binding affinity (Ka), stoichiometry (n), and enthalpy change (ΔH) associated with the peptide-mineral binding. Furthermore, the binding affinity is determined by analyzing the binding isotherm generated by titrating the peptide into the mineral solution. The stoichiometry of the binding reaction is determined via the ratio of the number of moles of the peptide to the number of moles of the mineral. The enthalpy change is obtained from the shape of the binding isotherm and thus provides information about the nature of the peptide-mineral interactions, e.g., hydrogen bonding, electrostatic interactions, and hydrophobic interactions.

SDS-PAGE (Sodium Dodecyl Sulfate Polyacrylamide Gel Electrophoresis) is a technique used to separate proteins or peptides based on their size. In peptide-mineral binding studies, SDS-PAGE is used to analyze the binding of a peptide to a mineral surface. The peptide sample is first loaded onto the gel, and then the mineral surface is added to the gel. After electrophoresis, the gel is stained to visualize the peptide bands. Therefore, a decrease in the intensity of the peptide band on the gel after mineral binding indicates that the peptide has bound to the mineral surface. However, it is important to note that SDS-PAGE is a semi-quantitative technique and may not provide accurate quantitative data on the degree of the peptide-mineral binding [67].

Fourier transform infrared spectroscopy (FTIR) is a technique used to study the chemical bonds present in a sample. In peptide-mineral binding studies, FTIR is used to identify the functional groups present in the peptide and the mineral surface and to study the interaction between them. FTIR works by measuring the absorbance of infrared radiation via the sample at different frequencies. Moreover, different functional groups absorb infrared radiation at different frequencies, which is further used to identify the presence of specific chemical bonds in the sample. In peptide-mineral binding studies, FTIR is also being used to study the bonding between the peptide and the mineral surface. FTIR works efficiently to study the interaction between the carboxyl groups on the peptide and the mineral surface or the interaction between the amino groups on the peptide and the mineral surface. For instance, in rice protein binding with cadmium, it showed that the location of the peaks was almost identical between the rice protein and the rice protein-cadmium complexes. The study demonstrated that the binding of cadmium to the rice proteins was primarily due to the non-covalent interactions between the complexes (Figure 2C) [80].

Mass spectrometry (MS) is a powerful technique that is used to study the interaction of peptides with minerals. MS provides information about the composition, structure, and binding properties of peptides and their complexes with minerals. Also, MS is used to identify the amino acid residues that interact with the mineral surface. In particular, matrix-assisted laser desorption/ionization (MALDI) MS can be used to detect peptides bound to mineral surfaces by analyzing the mass spectra of the mineral-bound peptides. Furthermore, by comparing the mass spectra of the mineral-bound and unbound peptides, the amino acid residues that are involved in the mineral-binding interactions are identified. For instance, a study used MALDI TOF/TOF to identify the peptides that chelate zinc (Figure 2D). Although the peptides were purified via macroporous adsorption resin, they still contained a variety of peptides. The two major peptides were selected for MS/MS analysis, which allowed for the determination of their sequences from the N to C terminus. The sequences were found to be Asn-Ala-Pro-Leu-Pro-Pro-Pro-Leu-Lys-His and His-Asn-Ala-Pro-Asn-Pro-Gly-Leu-Pro-Tyr-Ala-Ala. To study the binding efficiency of the two major zinc-chelating peptides, they were further synthesized. MS can be used in combination with other techniques, such as NMR and X-ray crystallography, to provide a more complete understanding of peptide-mineral interactions. On the computational side, molecular dynamics simulations, docking, and quantum mechanics/molecular mechanics (QM/MM) calculations can provide insight into the binding mechanism at the atomic level, including the energetics and dynamics of the interaction. Together, these techniques provide a comprehensive understanding of protein-mineral binding.

In conclusion, the use of various analytical techniques such as IMAC, RP-HPLC, ITC, SDS-PAGE, FTIR, and mass spectrometry provides valuable information about the binding affinity, mechanism, and thermodynamics of peptide-mineral complexes. These techniques help to identify the specific amino acid residues involved in the binding process, visualize the three-dimensional structure of the peptide-mineral complexes, and determine the functional groups involved in the interaction. While each technique has its advantages and limitations, their combined use can provide a comprehensive understanding of the peptide-mineral binding interaction. Together, these techniques provide a comprehensive understanding of protein-mineral binding.

## 4. Toxic Effects of Inorganic Minerals

### 4.1. Mechanism of Toxicity

Inorganic salts, such as calcium chloride, iron sulfate, zinc oxide, and magnesium chloride, have been utilized for their significant nutritional value and effectiveness in fortifying and supplementing food products. Although fortification using inorganic minerals was initially implemented as a beneficial approach to address micronutrient deficiencies, it has been associated with adverse health effects ranging from acute to chronic toxicity. For example, ferrous sulfate, an iron salt, commonly induces toxicity in the form of vomiting and nausea, which results from the accumulation of free radicals in the mucosal lining of the gastrointestinal tract surrounding the lumen [81]. The consumption of ferrous sulfate has been shown to increase the population of methanogenic species in the gut microbiota, which slows the gastrointestinal transit time and causes constipation [82]. In addition, ferrous fumarate intake can also lead to inflammation in the intestinal mucosa due to several factors, such as an increase in iron in the gastrointestinal tract, damage to the tight junction proteins present between the mucosal and enterocytes, and the reduction in the height, shape, and stability of intestinal villi [83]. This resulted in the impairment of nutrient absorption and other gastrointestinal issues [84].

**Figure 3:**
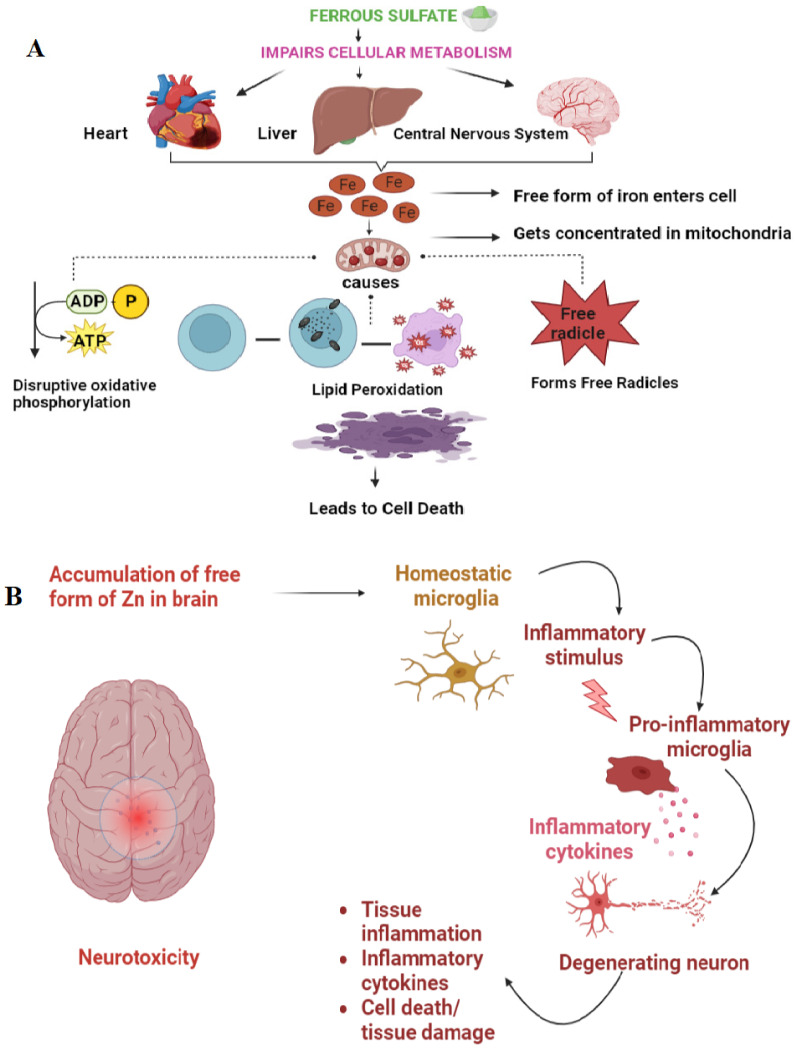
(**A**) Toxic effect of ferrous sulfate in the human body. (**B**) Neurotoxicity is caused by the accumulation of free form of zinc in the brain.

Iron-fortified food, such as ferrous fumarate-fortified maize porridge, has been found to exert a toxic effect on the gut microbiota. The concentration of unabsorbed inorganic minerals, specifically ferrous fumarate, in the gut lumen increases, promoting the growth of harmful enteropathogens and reducing the population of beneficial bacteria, such as Lactobacilli [85]. This imbalance in the gut microbiota leads to diarrhea [86]. Toxicity also arises from the accumulation of excess iron salts in the free, unbound form in both the blood and cells of the human body. This excess iron damages the plasma membrane and intracellular organelles via the reaction with hydrogen peroxide, forming reactive oxygen species that cause cellular changes (Figure 3A) [87].

The consumption of iron-fortified cereals increases the concentration of iron in the human body, which leads to toxic effects on the heart, including an increased risk of myocardial infarction and coronary artery disease [88]. This is because iron can overload the capacity of transferrin, which is an iron-binding protein, causing the release of free iron into the bloodstream [89]. The excess free iron damages vascular endothelial cells, which further contributes to the development of myocardial infarction and coronary artery diseases [90]. The consumption of excessive iron via the oral route may result in toxic effects, thereby elevating the possibility of developing colorectal cancer. This phenomenon can be attributed to the presence of free iron in human fecal matter, which promotes the formation of free radicals leading to cellular injury and subsequently increasing the risk of cancer [91]. Moreover, excess zinc salt can disrupt the functioning of enzymes in the brain, leading to several pathological conditions such as mitochondrial impairment, which is a major cause of the development of neurotoxicity (Figure 3B). In addition, excessive zinc salt can also cause neuroinflammation, which occurs due to the accumulation of cytosolic reactive oxygen species and the inhibition of mitochondrial energy production [92]. Iron toxicity can indeed affect the brain and lead to neurodegenerative disorders like Parkinson’s disease. Additionally excess iron can accumulate in the substantia nigra, a part of the midbrain that plays a key role in movement control, and this excess iron causes damage to dopaminergic neurons, which produces the neurotransmitter dopamine. The accumulation of iron in the brain can also cause oxidative stress and inflammation, which contribute to the development of Parkinson’s disease [93].

The presence of free Iron salts in the digestive system can have an inhibitory effect on the absorption of dietary iron. This is because free iron ions bind with antinutrients such as phytates and oxalates which are commonly found in various food sources, forming insoluble complexes that prevent the absorption of dietary iron by the body [94]. This leads to iron deficiency even in the presence of iron-rich foods, especially in populations with diets high in these antinutrients [95]. Carotenoids serve as antioxidants that safeguard the human body against chronic diseases. However, their absorption is hindered by the elevated levels of inorganic minerals, such as anhydrous magnesium chloride and calcium chloride, which have toxic effects. These minerals also reduce the bioaccessibility of various carotenoids by precipitating and interacting with unconjugated bile salts and free fatty acids. In addition to this, the bioaccessibility of carotenoids is further impacted by the decrease in macro viscosity and the absolute zeta potential of mixed micelles, along with the increase in surface tension [96]. Inorganic minerals not only impair the absorption of carotenoids but also interfere with the uptake of other minerals in the human body. For instance, excessive levels of zinc salt can reduce the absorption of copper due to the competitive absorbing mechanism of both minerals within enterocytes, which is mediated via metallothionein, a type of protein that binds to both copper and zinc. As a result, the bioavailability of copper decreases in the presence of an excess of zinc salt [97]. In addition, olfactory and oral sensations also develop a metallic taste upon oral iron consumption, which can contribute to the development of nausea.

Overall, inorganic salts such as calcium chloride, iron sulfate, zinc oxide, and magnesium chloride have been widely used to supplement and fortify food products due to their significant nutritional value. However, iron salts, such as ferrous sulfate and ferrous fumarate, cause gastrointestinal issues and impair the absorption of nutrients while also increasing the risk of heart disease, colorectal cancer, and neurodegenerative disorders. Similarly, excessive zinc salt can lead to neurotoxicity and neuroinflammation. Inorganic minerals can also interfere with the absorption of carotenoids and other minerals in the human body, leading to their reduced bioavailability. Therefore, it is important to carefully monitor the intake of inorganic minerals via fortified food products to prevent acute as well as chronic toxicity and promote optimal health.

### 4.2. In Vitro Studies: Toxicity Determination

Different assays are used, such as MTT (3-[4,5-dimethylthiazol-2-yl]-2,5 diphenyl tetrazolium bromide) and Glutathione, to analyze the toxicity of inorganic minerals. For instance, a cell line study that used SHSY5Y cells shows cell shrinkage, poor adhesion, and a loss of membrane integrity due to the excess accumulation of ferrous ions in the cell. This study also showed reduced cell viability due to oxidative stress and cell death in response to the higher concentration of iron in the cell culture. Moreover, the presence of an excess of free iron triggered the generation of reactive oxygen species and led to cell edema, characterized by cell swelling [98]. An excess of ferrous ions also has a toxicological effect on the RBC (red blood cell) parameters such as it causes changes in the morphology of the cell, hemoglobin derivatives, and stiffness in the membrane. The morphological changes observed in RBCs are a consequence of excess free iron, which reacts with hydrogen peroxide to generate highly reactive radicals. These radicals can cause damage to the lipids and proteins in the RBC membrane, leading to alterations in the nano-surface of the membrane and the cytoskeleton of the cell, ultimately changing its morphology. Furthermore, the accumulation of free ferrous ions increases the damage to packed red blood cells and induces membrane stiffening [99].

In a recent study utilizing neuronal PC12 cells, the administration of zinc salts induced several toxic effects, including a reduction in adenosine triphosphate and glutathione levels, resulting from an excessive influx of zinc ions into the cell. Moreover, the excess zinc ions entering the neurons led to toxic effects that triggered neuronal cell death. Cytotoxicity was observed at varying degrees with different zinc ions at different concentrations. Zinc citrate and zinc sulfate elicited the most significant impact, while zinc orotate, zinc acetate, zinc chloride, and zinc gluconate demonstrated moderate toxicity. Zinc histidine, on the other hand, exhibited toxic effects at the lowest concentration [100]. Zinc also showed genotoxicity due to the formation of mutagenic cells and damage caused to leukocyte DNA when the free form of zinc at high concentrations forms reactive oxygen species that react with nucleobases in DNA, causing oxidation and damage to the DNA structure. Similarly, in the intestinal line of CaCo-2 cells, the high concentration of zinc sulfate has been shown to cause toxic effects in the epithelial cells. The toxicity of iron in ferrous form caused a decrease in CaCo-2 cell viability and cell proliferation due to an increase in ferrous ions. The free form of ferrous ions also damaged the membrane stability because of their ability to generate free radicles and induce lipid peroxidation. The in vitro investigation of the toxicological impact of magnesium sulfate revealed cytotoxicity using an AGS cell line, which resulted in the inhibition of cell viability and proliferation. Additionally, magnesium sulfate was shown to affect the stomach by inducing the release of pro-inflammatory cytokines. An in vitro toxicological study of copper chloride showed cytotoxicity and genotoxicity through the cells of the anemone *Bunodosoma capsicum*. Cytotoxicity is caused by the reduction in cell viability as well as the cell number. Also, as the copper concentration increased with an increase in time, it induced oxidative stress and generated reactive oxygen species, thereby affecting the integrity of the cell membrane. Additionally, the increase in DNA fragmentation was observed due to the damage caused by copper chloride. The generation of reactive oxygen species via copper chloride caused genotoxicity and led to DNA degradation, which indirectly or directly induced oxidation by damaging lipids and proteins [101].

MTT and Glutathione assays analyze the toxicity of inorganic minerals on various cell lines. The excess accumulation of minerals in the cells resulted in various toxic effects such as oxidative stress, cell death, and morphological changes in red blood cells. Zinc, copper chloride, and magnesium sulfate are shown to cause cytotoxicity and genotoxicity and affect the release of pro-inflammatory cytokines. Thus, it highlights the significance of studying the toxicity of inorganic minerals in different cell lines to understand their harmful effects.

### 4.3. In Vivo Studies: Toxicity Determination

**Figure 4:**
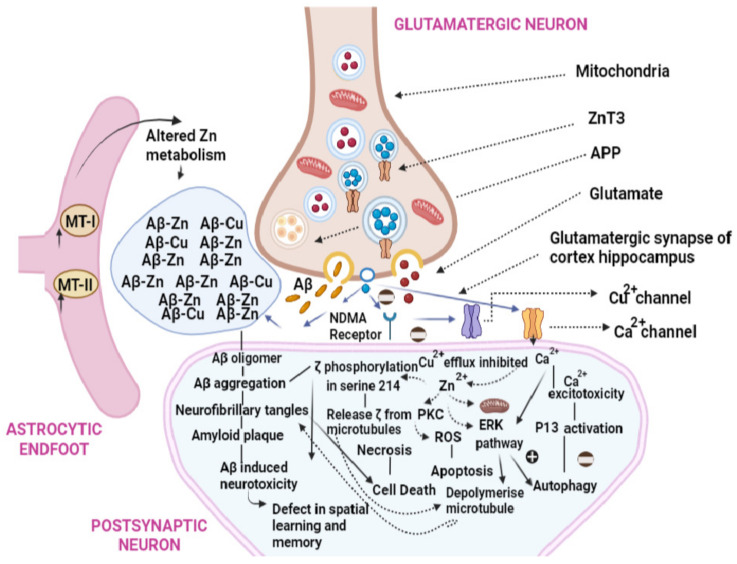
Mechanism of cognition dysfunction of the brain caused by zinc sulfate.

Several in vivo studies were conducted on rats and mice to analyze the effects of iron, zinc, and copper salts on different organs such as the lungs, liver, kidneys, and brain. In vivo studies conducted on the toxicological effects of iron salts using a rat model revealed that the presence of iron oxide resulted in adverse inflammation and fibrosis in the lungs. Furthermore, the presence of iron oxide in the lumen of alveolar capillaries impacted the body weight, liver, and kidney of both low-dose (LD) and high-dose (HD) rats. The observed mechanism of toxicity in rats was attributed to oxidative stress which was caused by iron oxide. A study on mice for the toxic effects of zinc sulfate showed that excess zinc sulfate causes cognition dysfunction and enhances the aggregation of β-amyloid (Figure 4). Moreover, the aggregation of β-amyloid was caused by the high concentrations of zinc sulfate, which altered the expression levels of the amyloid precursor protein and cleaved the enzymes in vivo. In the zinc-treated mice, the high concentration of zinc sulfate increased the number and size of the β amyloid immunoreactive senile plaques in the cortex and hippocampus part of the brain.

A study on rat liver for the analysis of the toxicity of copper chloride showed that the high levels of dietary copper caused mitophagy, apoptosis, and mitochondrial damage. This damage occurred due to the increased levels of reactive oxygen species which altered the morphology of mitochondria and induced swelling. Furthermore, the accumulation of copper chloride in the liver disrupted the pathways associated with mitophagy and apoptosis [102]. In rat liver mitochondria, the presence of copper and iron ions causes mitochondrial dysfunction due to the peroxidation of phospholipid and protein oxidation. Mitochondrial dysfunction includes a decrease in the rates of mitochondrial respiration, an alteration in the normal functioning of mitochondria, and cell death. An in vivo study on the effect of ferrous sulfate on the rat brain showed toxic effects such as lowered motor activity and behaviors that were related to anxiety. The presence of ferrous sulfate in the brain showed a decrease in locomotor activity and changes in reactivity to environmental stimuli. Also, iron overload affects the white blood cell count and corpuscular volume values by increasing their concentration and activating reactive oxygen species, which leads to oxidative stress [103]. The analysis of the effect of zinc succinate on male Wistar rats showed numerous toxic effects on various tissues. Specifically, the accumulation of zinc succinate in the cerebral cortex resulted in harm to neurons. Also, in the liver of rats, several sites with a focal necrosis of liver cells and periportal lymphohistiocytic infiltrate were observed. Toxicity was also observed in cardiac tissue, with an increase in dystrophic and cell alteration in the muscle of the heart along with the fragmentation of cardiomyocytes [104].

The reviewed studies have demonstrated that exposure to excessive amounts of inorganic minerals, including iron, zinc, and copper, can show toxic effects on various organs in rats and mice. The mechanism of toxicity is attributed to oxidative stress, mitochondrial dysfunction, and altered expression levels of specific enzymes and proteins. The observed toxic effects include inflammation and fibrosis in the lungs, cognition dysfunction, β-amyloid aggregation in the brain, mitophagy, apoptosis, and mitochondrial damage in the liver, lowered motor activity and behaviors related to anxiety in the brain, and harm to neurons, liver cells, and cardiac tissue. These findings highlight the importance of regulating the intake of inorganic minerals to avoid potential health risks associated with an inorganic minerals overload.

### 4.4. Sensory Challenges of Inorganic Minerals

Moreover, inorganic salts have also imposed many challenges when added to different types of food (milk, cereals, juices, and cookies) and have exhibited numerous challenges by causing changes in sensory attributes, making them unfit for human consumption. For instance, in a study, several inorganic minerals were assessed for their sensory characterization, namely calcium chloride, lactate, glycerophosphate; ferrous sulfate, chloride, gluconate; magnesium sulfate and chloride; and zinc sulfate and chloride. It was observed that these compounds imparted a metallic taste, astringency, glutamate sensation, and bitterness, thereby consequently compromising their acceptability. Many calcium salts also affect acceptability by increasing the chalkiness, acidity, bitterness, and changes in the flavor of food [105]. Also, the interaction of soluble calcium salts with other food components (tannins and anthocyanins) causes the darkening and red-to-blue color in food. The addition of calcium malate to a beverage for fortification purposes increased the astringency, affecting the taste profile of the beverage.

Studies have demonstrated that the use of calcium salts, such as calcium sulfate and calcium carbonate, in the fortification of a flat-type bread can lead to a chalky taste, bitter mouthfeel, and throat-catching issues. Inorganic calcium minerals have also been found to induce sensory changes in food products due to the increased crosslinking of the food matrix. For example, the fortification of cheese, yogurt, and milk with calcium salts such as calcium lactate gluconate, tribasic calcium phosphate, and calcium carbonate can result in cross-linking with pectins and phosphate, leading to sedimentation and rendering the food product, thus making it unsuitable for functional use. Moreover, the use of inorganic minerals of iron such as ferrous chloride, ferrous sulfate, and ferrous gluconate showed a strong metallic taste. Additionally, among the three inorganic minerals, ferrous gluconate has been observed to produce the highest levels of astringency and sourness. Nonetheless, inorganic minerals also affect the physical properties of food along with sensory properties. For instance, the fortification of zinc sulfate in cookies affected their physical structure by reducing the diameter and thickness of cookies [106]. Also, in a study conducted on the preparation of fortified chickpea flour, the inorganic minerals induced lipid oxidation, leading to rancidity, and ultimately, an undesirable change in color in the food [107]. In a zinc-based fortified food, the addition of zinc sulfate has been found to reduce the flavor attribute of bakery products. Similarly, the fortification of cheese with zinc sulfate has been linked to hardness and an off-flavor. On the other hand, fortified cheese using ferrous sulfate to increase its iron content has been associated with a metallic taste. In the fortification of goat milk, an intense off-taste and a strong acetic flavor were observed because of magnesium acetate and magnesium chloride bis-glycinate salts [108].

Therefore, it is crucial to consider the sensory and physical attributes of food during the process of fortification with inorganic minerals, to guarantee that the fortified food is not only nutritionally valuable but also appealing and acceptable to the consumers. Furthermore, it is suggested that alternative methods, such as mineral binding with proteins derived from cereals and legumes, be investigated as a means of ensuring adequate intake without the possibility of toxicity.

## 5. Application of Cereal- and Legume-Derived Protein-Mineral Complexes

### 5.1. Fortification of Food Products

Protein-mineral complexes derived from cereals and legumes have found extensive applications in fortifying food products, particularly in addressing global malnutrition issues. These complexes can be enriched with essential minerals like iron, zinc, calcium, and vitamins. When incorporated into various food items such as cereals, bread, pasta, and dairy alternatives, they serve as an effective means to combat nutrient deficiencies, especially in regions where access to diverse and nutritious foods is limited [109,110,111]. For instance, iron-fortified cereal products have been pivotal in alleviating iron-deficiency anemia, a prevalent health concern worldwide. Additionally, these complexes can be tailored to release nutrients gradually during digestion, ensuring optimal absorption and bioavailability [112].

### 5.2. Enhancing Nutritional Value

The different applications of cereal- and legume-based protein-mineral complexes are related to several improved functional effects such as the increase in antioxidant properties, enhanced bone mineral density in vivo, and decreased deficiency of iron in vitro, and future recommendations should focus on the utilization of these complexes as a food ingredient for both dietary and nutraceutical supplementation of mineral for future research. Moreover, the hydrolyzed red lentil protein + iron complexes in vitro decreased the expression levels (DMT—Divalent metal transporter 1, TFR—Transferrin receptor, and ANKRD—Ankyrin repeat domain 37 mRNA) which were induced due to iron deficiency anemia. The decrease in the anemic condition in Caco-2 cells was due to the protein-iron complexes treatment which was supplied to this cell culture system [113]. Also, some protein-mineral complexes served antioxidant functionality due to their strong mineral binding and chelating effects. The major benefit of antioxidant functionality is it helps in protecting the body against oxidative stress and reduces the risk of chronic diseases. Antioxidants are compounds that neutralize the free radicles in the body, which are harmful molecules that damage the cells and tissues. Thus, increasing the functionality of antioxidants’ cereal- and legume-based protein-mineral complexes may help prevent or mitigate the damage caused by the radicle.

For instance, the mung bean protein + iron complexes showed antioxidant function in vitro, and the hydrolyzed chickpea protein + copper complexes also demonstrated a similar functionality [114]. Moreover, protein-mineral complexes have shown promising effects in providing health benefits to humans. Peanut protein + calcium complexes when supplied to male mice in vivo improved the bone structure, promoted calcium absorption, and also enhanced the bone mineral density with no adverse side effects [66]. Therefore, providing a source of calcium that is easily absorbed by the body, peanut protein + calcium complexes may help to prevent or mitigate the effects of calcium deficiency.

A lot of studies on cereal- and legume-based protein-mineral complexes suggested that with a wide range of positive effects on human health in eradicating deficiencies, these can be used as an approach for the future in developing novel dietary and nutraceutical supplements at the industrial level [69].

## 6. Conclusions

In conclusion, the issue of micronutrient deficiencies is a global concern that affects individuals across various age groups, with pregnant women and young children being particularly vulnerable. The deficiencies of essential nutrients like iron, zinc, calcium, magnesium, and copper can have profound and far-reaching health consequences. These deficiencies disrupt vital physiological processes, impair immune function, hinder cognitive development, and increase susceptibility to infections, among other adverse effects. Recognizing the gravity of the problem, there has been a growing acknowledgment of the need to address micronutrient deficiencies by governments, non-governmental organizations, and the food industry. However, diversifying diets to meet nutritional needs is not always feasible, and certain foods may contain anti-nutritional components that hinder mineral absorption. As a response to this challenge, food fortification with inorganic mineral sources has emerged as a viable solution. These inorganic salts have been used for many years to fortify a wide range of food products, providing an efficient and cost-effective means of improving the nutritional status of populations at risk of deficiencies. However, recent research has shed light on the potential drawbacks of relying solely on inorganic minerals for fortification. One of the major concerns is the toxicity associated with the long-term use of inorganic minerals. The accumulation of these minerals in the body can lead to a range of health issues, from mild discomfort to severe conditions affecting the liver, brain, and metabolic balance. The release of reactive oxygen species via free inorganic minerals can further exacerbate the toxicity concerns. To address these issues, recent studies have explored the utilization of mineral-protein interactions to form complexes with inorganic minerals. This innovative approach holds promise in enhancing the bioavailability and safety of mineral fortification, reducing the risk of toxicity, and ensuring the efficient utilization of these essential nutrients in the human body.

## Figures and Tables

**Table 2:** Binding efficiency of different protein-mineral complexes.

Protein and Mineral Interaction	Mineral Salt	Binding Efficiency	References
Peanut protein isolate + Zinc	Zinc Sulfate	124.7 mg/g	
Barley protein hydrolysate + Iron	Ferrous Chloride	188 µg/mL	[39]
Barley protein hydrolysate+ Calcium	Calcium Chloride	141 µg/mL	[39]
Barley protein hydrolysate+ Copper	Copper Sulfate	134 µg/mL	[39]
Barley protein hydrolysate+ Zinc	Zinc acetate	125 µg/mL	[39]
Soybean protein hydrolysate + Iron	Ferrous Chloride	3.87 mg/g	[43]
Soybean protein hydrolysate + calcium	Calcium chloride	66.9 mg/g	[45]
Jamapa protein hydrolysate + Iron	Ferrous Chloride	15.68%	[45]
Jamapa protein hydrolysate + Copper	Copper Sulfate	81.63%	[45]
Black bean protein hydrolysate + Calcium	Calcium chloride	77.54 µg/mg	[43]
Red lentil protein hydrolysate + Iron	Ferrous sulfate heptahydrate	98%	[48]
Mung bean protein hydrolysate + Iron	Ferrous chloride tetrahydrate	153.59 mg/g	[41]
Oat protein hydrolysate + Iron	Ferric chloride	39.7%	[34]
Mung Bean protein hydrolysate + Iron	Ferrous sulfate heptahydrate	61.25 µg/mg	[44]
Soybean protein hydrolysate + Iron	Ferric chloride	12.42 mg/g	[37]
Sunflower seed protein + Calcium	Calcium chloride	124.7 mg/g	[43]
Mung bean protein + Calcium	Calcium chloride	196 mg/g	[41]
Mung bean protein + Iron	Ferrous chloride tetrahydrate	19.24 mg/g	[41]
Rice Protein + Cadmium	Cadmium chloride	15.26 mg/g	[55]
Wheat Germ Protein Hydrolysate + Calcium	Calcium chloride	67.5 mg/g	[59]

## Data Availability

The data are available within the article.
